# Supplementary material for: Identification of photoperiod-induced specific miRNAs in the adrenal glands of Sunite sheep (Ovis aries)
Source: Front Vet Sci. 2022 Jul 22;9:888207. doi: 10.3389/fvets.2022.888207 (PMC9354845; doi:10.3389/fvets.2022.888207)
Supplement: Supplementary file 1 [file Data_Sheet_1.ZIP › Supplementary file/miRDeep2 file.zip!/miRDeep2 file/Know_Structure/LP42A3/oar-mir-495.pdf]

miRBase precursor : oar-mir-495
Total read count : 27807
oar-miR-495-5p read count : 138
oar-miR-495-3p read count : 27637
remaining reads : 32

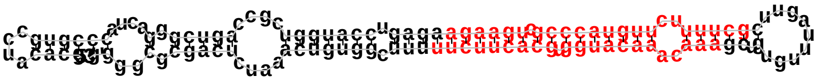

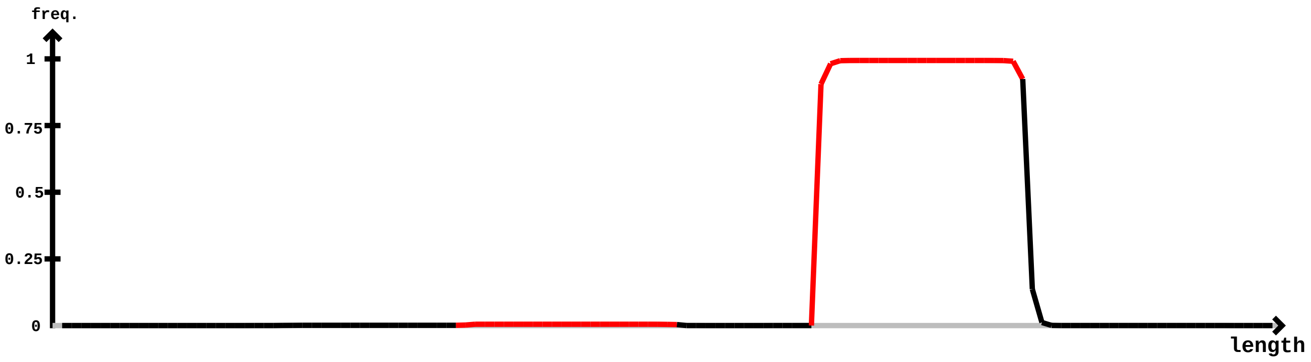


oar-miR-495-3p

oar-miR-495-5p

```
5'- gcacauccgugcccaucagggcugaccgcugguaccugagaagaagucgcccauguucuuuucgcuugauuugugucgaaacaaacauggugcacuucuuuuucggugucaaaucucagcgcgggg  -3'  exp
    ((((....))))((....(.(((((....(((((((.((((((((((.(((((((((..((((((.........).))))).))))))).))))))))))).)))))))....))))).)..))   reads   mm
    ..........gcccaucagggcugaccgcugguaccugag......................................................................................   1       0
    ...................ggcugaccgcugguaccugagaa....................................................................................   1       0
    .....................cugaccgcugguaccugagaa....................................................................................   1       0
    ......................ugaccgcugguaccugagaa....................................................................................   10      0
    .......................gaccgcugguaccugagaa....................................................................................   6       0
    ........................accgcugguaccugaga.....................................................................................   1       0
    ........................accgcugguaccugagaa....................................................................................   8       0
    .........................................agaagucgcccauguucuuu.................................................................   1       0
    .........................................agaagucgcccauguucuuuu................................................................   5       0
    .........................................agaagucgcccauguucuuuuc...............................................................   5       0
    .........................................agaagucgcccauguucuuuucg..............................................................   18      0
    ..........................................gaagucgcccauguucuuu.................................................................   2       0
    ..........................................gaagucgcccauguucuuuu................................................................   8       0
    ..........................................gaagucgcccauguucuuuuc...............................................................   13      0
    ..........................................gaagucgcccauguucuuuucg..............................................................   82      0
    ..........................................gaagucgcccauguucuuuucgc.............................................................   1       0
    ..........................................gaagucgcccauguucuuuucgcu............................................................   3       0
    ......................................................................uugugucgaaacaaacauggugcacuucuu..........................   1       0
    .........................................................................ugucgaaacaaacauggugcacuucuu..........................   1       0
    ..............................................................................aaacaaacaCggugcacu..............................   1       1
    ..............................................................................aaacaaacauggugcacuA.............................   1       1
    ..............................................................................aaacaaacauggugcacuu.............................   9       0
    ..............................................................................aaacaaacauggugcacuuc............................   60      0
    ..............................................................................aaacaaacauggugcacuucu...........................   1714    0
    ..............................................................................aaacaaacauggugcacuucuu..........................   20921   0
    ..............................................................................aaacaaacauggugcacuucuuu.........................   2399    0
    ..............................................................................aaacaaacauggugcacuucuuuu........................   64      0
    ..............................................................................aaacaaacauggugcacuucuuuuu.......................   3       0
    ...............................................................................aacaaacauggugcacuuc............................   8       0
    ...............................................................................aacaaacauggugcacuucu...........................   138     0
    ...............................................................................aacaaacauggugcacuucuu..........................   941     0
    ...............................................................................aacaaacauggugcacuucuuu.........................   975     0
    ...............................................................................aacaaacauggugcacuucuuuu........................   61      0
    ................................................................................acaaacauggugcacuucu...........................   5       0
```

```
                                                                           oar-miR-495-3p
                                         oar-miR-495-5p
gcacauccgugcccaucagggcugaccgcugguaccugagaagaagucgcccauguucuuuucgcuugauuugugucgaaacaaacauggugcacuucuuuucggugucaaaucucagcgcgggg

................................................................................acaaacauggugcacuucuu.........................   44   0
................................................................................acaaacauggugcacuucuuu........................   98   0
................................................................................acaaacauggugcacuucuuuu.......................   169  0
................................................................................acaaacauggugcacuucuuuuu......................   4    0
.................................................................................caaacauggugcacuucuu.........................   1    0
.................................................................................caaacauggugcacuucuuu........................   1    0
.................................................................................caaacauggugcacuucuuuu.......................   9    0
.................................................................................caaacauggugcacuucuuuuu......................   9    0
..................................................................................aaacauggugcacuucu..........................   1    0
..................................................................................aaacauggugcacuucuuu........................   1    0
....................................................................................................uucggugucaaaucucagcg.....   2    0
```
